# PHYSICAL FITNESS MODERATES THE AGE-RELATED ASSOCIATION BETWEEN EXECUTIVE FUNCTIONING AND MOBILITY

**DOI:** 10.1093/geroni/igac059.2386

**Published:** 2022-12-20

**Authors:** Emma Dupuy, Florent Besnier, Christine Gagnon, Juliana Breton, Catia Lecchino, Mathieu Gayda, Louis Bherer

**Affiliations:** Université de Montréal, Montreal, Quebec, Canada; Université de Montréal, Montreal, Quebec, Canada; Montreal Heart Institute, Montreal, Quebec, Canada; Montreal Heart Institute, Montreal, Quebec, Canada; Montreal Heart Institute, Montreal, Quebec, Canada; Université de Montréal, Montreal, Quebec, Canada; University of Montreal, Montreal, Quebec, Canada

## Abstract

In older adults, executive functions are important for daily-life function and mobility. Evidence suggests that the relationship between cognition and mobility is dynamic and could vary according to individual factors, but whether cardiorespiratory fitness reduces the age-related increase of interdependence between mobility and cognition remains unexplored. One hundred eighty-nine participants (aged 50-87) were divided into three groups according to their age: middle-aged (MA; < 65), young older adults (YOA; 65-74), and old older adults (OOA; ≥75). Participants performed Timed Up and Go and executive functioning assessments (Oral Trail Making Test and Phonologic verbal fluency) remotely by videoconference. Participants completed the Matthews questionnaire to estimate their cardiorespiratory fitness (VO2 max in ml/min/kg). A three-way moderation was used to address whether cardiorespiratory fitness interacts with age to moderate the relationship between cognition and mobility. Results showed that the cardiorespiratory fitness x age interaction moderated the association between executive functioning and mobility (β = -.05, p = .047) (R2 = .18, p <.0001). At lower levels of physical fitness (< 19.16 ml/min/kg), executive functioning significantly influenced YOA’s mobility (β = -.48, p = .004) and to a greater extent OOA’s mobility (β = -.96, p = .002). Our results support the idea of a dynamic relationship between mobility and executive functioning during aging and suggest that physical fitness could play a significant role in reducing their interdependency.

